# Deficits in Degraded Facial Affect Labeling in Schizophrenia and Borderline Personality Disorder

**DOI:** 10.1371/journal.pone.0154145

**Published:** 2016-06-14

**Authors:** Annemiek van Dijke, Mascha van ‘t Wout, Julian D. Ford, André Aleman

**Affiliations:** 1 Yulius-academy/ Yulius-COLK, Rotterdam-Dordrecht Area & Department of Clinical Psychology, VU University Amsterdam, Department of Psychiatry, Academic Medical Center, Amsterdam, the Netherlands; 2 Department of Psychiatry and Human Behavior, Alpert Medical School of Brown University, Providence, United States of America; 3 Department of Psychiatry, University of Connecticut Health Center, Farmington, CT, United States of America; 4 Department of Neuroscience, University Medical Center Groningen, and Department of Psychology, University of Groningen, Groningen, the Netherlands; Central Institute of Mental Health, GERMANY

## Abstract

Although deficits in facial affect processing have been reported in schizophrenia as well as in borderline personality disorder (BPD), these disorders have not yet been directly compared on facial affect labeling. Using degraded stimuli portraying neutral, angry, fearful and angry facial expressions, we hypothesized more errors in labeling negative facial expressions in patients with schizophrenia compared to healthy controls. Patients with BPD were expected to have difficulty in labeling neutral expressions and to display a bias towards a negative attribution when wrongly labeling neutral faces. Patients with schizophrenia (N = 57) and patients with BPD (N = 30) were compared to patients with somatoform disorder (SoD, a psychiatric control group; N = 25) and healthy control participants (N = 41) on facial affect labeling accuracy and type of misattributions. Patients with schizophrenia showed deficits in labeling angry and fearful expressions compared to the healthy control group and patients with BPD showed deficits in labeling neutral expressions compared to the healthy control group. Schizophrenia and BPD patients did not differ significantly from each other when labeling any of the facial expressions. Compared to SoD patients, schizophrenia patients showed deficits on fearful expressions, but BPD did not significantly differ from SoD patients on any of the facial expressions. With respect to the type of misattributions, BPD patients mistook neutral expressions more often for fearful expressions compared to schizophrenia patients and healthy controls, and less often for happy compared to schizophrenia patients. These findings suggest that although schizophrenia and BPD patients demonstrate different as well as similar facial affect labeling deficits, BPD may be associated with a tendency to detect negative affect in neutral expressions.

## Introduction

Facial affective expressions are informative about a person’s inner state and can yields information that may guide social interactions. Schizophrenia and borderline personality disorder (BPD) often involve severe social-emotional disturbances and problems with mentalizing or meta-cognitive functioning [1–3]. However, the similarities and differences of the cognitive-emotional underpinnings of these impairments are not well defined or documented, and require further scientific study.

Patients with BPD have been observed to fear negative social valuation leaving them vulnerable to experience abandonment and rejection [1, 4]. Additionally, patients with BPD are characterized by negative emotional states, maladaptive cognitive processes [5] and distinct impairments in emotion regulation resulting in affective instability which can be so severe as to result in hospitalization and suicide attempts (e.g. [6–8]). Patients with schizophrenia on the other hand have been reported to show a blunting or flattening of affect as well as social and emotional withdrawal, within the domain of negative psychotic symptoms. In addition, positive psychotic symptoms in schizophrenia may include diffuse distress and pervasive distorted beliefs about relationships, such as delusional ideas of reference in relation to other persons (e.g. [3, 9]).

Because the face is important for signaling emotions, especially in social situations, facial affect processing is commonly used as a measure of social-emotional functioning both in schizophrenia [10] and BPD [11–12]. Some studies report that BPD patients are less accurate in the recognition of negative facial expressions including anger, disgust, fear and sadness [13–15]. In contrast, other studies demonstrated that facial emotion detection might be enhanced in BPD [16], such as a tendency to ascribe an emotion when presented with neutral facial expressions [17]. This tendency to detect emotions in neutral or ambiguous faces may be specific for over-reporting negative emotions [18, 19], such as fear [20]. Moreover, BPD has been associated with lower ratings of happiness in low intensity happy facial expressions, together with a reduced confidence in their judgement of these faces [21].

A growing body of research also reports facial affect recognition deficits associated with schizophrenia (see for extensive reviews [22, 23]) and which specific for negative emotions such as fear [24, 25]. These impairments in social cognition have been associated with poor social functioning and outcome in schizophrenia [26–30].

Despite both schizophrenia and BPD being associated with difficulties processing negative social-emotional information, the specificity and similarities of negative emotion-processing deficits have not been directly tested. To our knowledge there are currently no studies that directly compare these two groups on facial affect processing in addition to comparison with a healthy control group. Furthermore, most studies do not include a psychiatric control group in order to test whether lower performance on a task can be ascribed to general psychiatric status or is specific for a given disorder. In the current study we opted to include patients with somatoform disorders (SoD) as a psychiatric control group.

SoD was selected because it appears to involve difficulties in the awareness and regulation of emotions; however the emotional difficulties are not necessarily social in nature as appears to be the case with both schizophrenia and BPD. For instance patients with SoD frequently display complaints about unexplainable physical symptoms thought to have an emotional basis [8, 30, 31]. Furthermore, SoD has been associated with difficulties in the ability to express and identify their own feelings [32–37] as well as with lower levels of emotional awareness [38–40]. The present study therefore extends the current literature by assessing facial affect labeling performance of happy, angry, fearful and neutral facial expressions in schizophrenia and BPD, including a psychiatric control group (SoD) as well as a healthy control group. The data reported here on schizophrenia patients and healthy controls have been published before [25, 41], and the current analyses extend those findings by providing comparisons on facial affect processing in BPD and SoD, as well as by examining differences in processing errors that may differentiate BPD and schizophrenia.

Facial affect labeling, the focus of the present study, in contrast to affect matching or discrimination, adds a verbal information processing component and involves explicit categorization which can be specifically important for social communication. Moreover, we used degraded facial affect stimuli as prior research has demonstrated that the processing of such stimuli might depend more on top-down processing from brain systems involved in attention, mental imagery and feature binding [42, 43]. We hypothesized that a tendency to interpret (ambiguous, i.e. neutral) faces as fearful or angry in BPD, as reviewed above (also [44]), would be more pronounced when using degraded stimuli that depend more on top-down processing. We further hypothesized, based on the reviewed literature above, that patients with schizophrenia would show more difficulties in the labeling of degraded negative facial affects compared to the non-psychiatric and psychiatric control group. We did not expect differences between SoD and the non-psychiatric control group on facial affect labeling.

The multiple-choice nature of our task further allowed examination of differences in misattribution errors between groups. We expected BPD patient to more often mislabel degraded neutral faces as negative (fearful or angry) than the other three groups ((non-psychiatric) controls and schizophrenia). Finally we tested whether the labeling of degraded facial affect was more disrupted when faces displayed lower emotional intensity as compared to full-blown emotional expressions. Lower emotional intensity might be more reflective of every-day situations in which emotions are often quick, mixed and not fully expressed. We hypothesized that all groups would benefit from greater emotional intensity, but that this benefit would be reduced for BPD and schizophrenia patients due to either a hypersensitivity to lower emotional intensity in detecting facial affect as observed in BPD [45] or a failure to benefit from greater emotional intensity as reported in schizophrenia [46]

## Methods and Materials

### Participants and procedures

In total 153 participants were included in this study by licensed clinicians using clinical interviews; 57 participants were diagnosed with schizophrenia (BPD or SoD were ruled out); 30 participants had a diagnosis of BPD (schizophrenia and SoD were ruled out); 25 patients received the diagnosis of SoD (schizophrenia and BPD were ruled out); and 41 participants with no mental disorder were included as a healthy control group.

All patients with a diagnosis of BPD and SoD were recruited from Delta Psychiatric Center, Poortugaal, the Netherlands and participated in a multicenter project on Clinical Assessment of Trauma-Related Self and Affect Dysregulation [47] as inpatients receiving three weeks of intense clinical psychotherapy group treatment, comparable to day hospital programs. This means that patients did not need assistance with independent living, but were impaired enough due to symptoms to justify an intense psychotherapy program. A history of potential brain damage and high doses of psychotropic medications that may impair executive function, other mental and developmental disorders (i.e., psychotic disorders (including schizophrenia), bipolar disorder, depression with imminent suicidality, eating disorders, autism spectrum disorders or attention deficit hyperactivity disorder) that would interfere with cognitive functioning were exclusion criteria. During intake trained and officially registered clinicians (psychiatrists, psychotherapists) assessed GAF as part of DSM diagnosis to be around a score of 50 for those with BPD and around a score of 45 for those with SoD to ratify the need for admission to inpatient psychotherapy group treatment (GAF data were unfortunately not accessible for statistical analysis).

Of the schizophrenia group 20 patients were recruited from Delta Psychiatric Center and 37 patients were recruited from the University Medical Center Utrecht, the Netherlands. Healthy control participants were recruited from the general population using advertisements in local newspapers. Exclusion criteria for all groups were neurological conditions affecting the central nervous system, a history of head injury with loss of consciousness, recent history of substance abuse and mental retardation. A history of having a mental disorder was an additional exclusion criterion for the control group. The local ethics committees approved the study and all participants provided written informed consent after the procedure had been fully explained, according to the Declaration of Helsinki.

#### Schizophrenia participants

Of the 57 patients diagnosed with schizophrenia, 35 were diagnosed with paranoid schizophrenia and 22 with non-paranoid schizophrenia. In the schizophrenia sub-sample, 30 patients were inpatients at the time of the study and 27 were outpatients. The medications they were receiving according to medical records were Antipsychotics (number of patients receiving medication): Clozapine (23); Risperidone (9); Olanzapine (6); Quetiapine (2); Sulpiride (1); Bromperidole (1); Pimozide (2); chloorprotixeen (1); zuclopentixol (1); Antidepressants: Paroxetine (2); Citalopram (2); Mood stabilizers: Lithium (1); Valproate (2); Benzodiazepines: Oxazepam (3); Temazepam (1); Lorazepam (3). 8.8% (5) of schizophrenia patients did not receive any medication. Before task administration the Positive and Negative Syndrome Scale (PANSS) [48] was administered for an indication of severity of schizophrenia symptoms by two trained raters (master-level clinical research assessors under supervision MvtW for evaluation of reliability of diagnosis). Average PANSS positive scale was 13.67 (SD 6.10); range 7–34; average PANSS negative scales was 13.07 (SD 4.86), range 7–34; average PANSS general psychopathology was 27.33 (SD 7.16), range 17–48. A DSM-IV diagnosis of schizophrenia was obtained by two trained raters, one rater always being a trained psychiatrist, administering the Comprehensive Assessment Symptoms and History (CASH) interview [49], with independent review and confirmation of the diagnoses by UMCU master-level clinical (research) staff or MvtW in all cases. Although presence of comorbidity was a strict exclusion criteria for this sample, seven inpatients reported a score on the Beck’s Depression Inventory [50] of 20 indicating moderate to severe depression (N = 2 out of 7).

#### Borderline Personality Disorder participants

The Borderline Personality Disorder Severity Index (BPDSI) [51], (Dutch version IV: [52, 53]) was administered (by AvD) to rate severity and verify DSM-IV diagnostic criteria of BPD and for all cases reliability of the rates was evaluated with clinical coworkers (psychiatrist/ clinical psychologists) to come to a consensus diagnoses of BPD. For inclusion in this study a cut-off score of 20 also was used and mean BPDSI (22 out of 30 participants) was 33.96 (SD 8.65), range 22–58. All patients included in the BPD sample were inpatients. The medications they were receiving according to medical records were Antipsychotics (number of patients receiving medication): Risperidone (5); Olanzapine (2); Quetiapine (1). Antidepressants: Paroxetine (4); Citalopram (5); Sertraline (3); Fluoxetine (1). Benzodiazepines: Oxazepam (3); Temazepam (1); Lorazepam (1). 40% (12 out of 30) of BPD patients did not receive any medication and about 50% of patients receiving medication were on 2 or more types of medication.

#### Somatoform disorder participants

The Composite International Diagnostic Interview (CIDI) [54], (Dutch version [55]) was administered (by AvD) to verify the DSM-IV diagnostic criteria of SoD and for all cases reliability of diagnosis was evaluated with clinical coworkers (psychiatrist/ clinical psychologists) to come to a consensus diagnosis of SoD. Excluded were participants with hypochondria; somatoform disorder NOS; and body dysmorphic disorder based on section C of the CIDI. Next, SoD diagnosis was confirmed (and somatic illness ruled out) by independent review by a specialist in internal medicine. Three groups of SoD patients were included; patients with somatoform disorder, undifferentiated somatoform disorder, and patients with a combined conversion disorder and pain disorder (not body dysmorphic disorder or hypochondria). All patients included in the SoD sample were inpatients. The medications they were receiving according to medical records were Antipsychotics (number of patients receiving medication): Risperidone (3); Quetiapine (5). Antidepressants: Paroxetine (6); Citalopram (3); Fluoxetine (2); Sertraline (2). Benzodiazepines: Oxazepam (3); Temazepam (1); Diazepam (1); Lorazepam (4). 20% (5 out of 25) of SoD patients did not receive any medication and about 40% of patients receiving medication were on 2 or more types of medication.

#### Healthy control participants

All controls were screened for the absence of mental disorders and drug use with the Mini International Neuropsychiatric Interview (M.I.N.I.) [56]. None of the control participants reported the presence of symptoms sufficiently indicating psychiatric disorders or the use of medication or drugs.

### Materials

#### Degraded Facial Affect Recognition task

**Fig 1:**
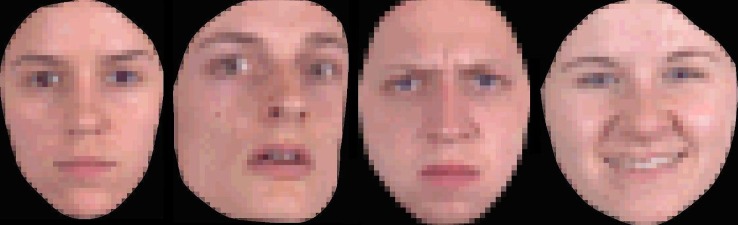
Example of degraded face stimuli (neutral; fearful; angry; happy, all 100% emotion intensity) used in the degraded facial affect labeling task.

This is a forced-choice facial affect labeling of degraded faces [25, 41, 57] programmed using E-Prime software [58]. Degradation of images was done to promote top-down processing influences from brain systems involved in attention, mental imagery and feature binding when processing these degraded faces [42, 43]. Moreover, low-frequency, reduced contrast (fearful) facial expressions have been associated with greater amygdala activity [59], a brain region important for emotional processing and associated with BPD [60, 61] as well as schizophrenia [62]. Degradation was obtained by passed photographs of faces through a filter that reduced visual contrast by 30%. Photographs of 4 different actors, 2 male and 2 female, were used from a previous developed and used set of faces [63]. Sixty-four trials were presented on a computer screen and consisted of 16 face presentations in each of 4 conditions: angry, happy, fearful, and neutral. For angry, fearful and happy expressions, 8 out of 16 trials displayed 100% emotional intensity and the other 8 trials displayed 75% emotional intensity as created from a morph. Emotional intensity (100% or 75%) refers to the amount of emotion presented in the picture, e.g. the 100% happy condition showed an obviously happy face, whereas happiness was less obvious in the 75% happy condition. This resulted in each angry, happy, fearful facial expression picture being presented twice (once with 75% emotional intensity and once with 100% emotional intensity) and each neutral facial expression was presented four times. See Fig 1 for an example of the stimulus used. Subjects were asked to indicate the expression of each face by forced-choice with button press (F1–F4) or by mouse. The labels (“angry,” “sad,” etc.) were presented at the bottom of the screen to remind subjects of the different emotion categories. No time limit was given and participants were asked to respond as accurately as possible.

#### General face recognition

The Benton Van Allen Test of Facial Recognition, short version [64] was administered to examine general face recognition ability. This test consists out of 13 trials requiring 27 responses in which participants are asked to compare a target face with six other faces and point which three of the six faces is the same as the target face.

### Statistical Analyses

The data were analyzed with SPSS, version 20 [65]. Differences between the four groups on general face recognition were tested using AN(C)OVA. Group differences on degraded facial affect labeling was tested using a 3(facial affect expression: happy; fearful; angry)x 2(emotional intensity: 100%; 75%)x4(group: controls; schizophrenia; BPD; SoD patients) GLM mixed design repeated measures AN(C)OVA. Neutral face stimuli were excluded from this analyses due to the lack of two levels of emotional intensity in these faces. Potential group differences on labeling neutral face stimuli were examined as part of subsequent follow-up analyses using MAN(C)OVA. In case of significant differences between the groups on demographic variables and the Benton Test of Facial Recognition these variables were considered as covariates when examining general face recognition and facial affect labeling respectively in the in the above-mentioned analyses. Alpha level was set at 0.05, two-tailed. In cases where sphericity cannot be assumed, a Greenhouse-Geisser correction was performed. Due to the number of analyses to be performed we report Bonferroni corrected (post-hoc) group comparisons on experimental tasks.

## Results

### Demographics

The groups differed significantly on age, *F*(3,152) = 3.13, *p* = 0.03. Post-hoc testing demonstrated that SoD patients are significantly older than all other groups (BPD: *p* = 0.005, schizophrenia: *p* = 0.04, controls: *p* = 0.01). Controls, BPD and schizophrenia patients did not differ significantly from one another (all *p*>0.05). The groups also differed significantly on sex, *Chi-square =* 35.43, *df* = 3, *p*<0.001. Even though the schizophrenia group and control group did not differ (*Chi-square =* 0.96, p = 0.33) and the SoD group did not differ from the BPD group (*Chi-square* = 2.16, *p* = 0.14), both the BPD and SoD group differed significantly from the control and schizophrenia group (all *Chi-square* > -16.06, all *p*< 0.05). The four groups also differed significantly on educational level (low; medium; high), Kruskal Wallis *Chi-square =* 10.69, *df* = 3, *p* = 0.01. Similar as before, the schizophrenia group and control group did not differ (Mann-Whitney *Z* = -0.49, *p* = 0.62) and the SoD group did not differ from the BPD group (Mann-Whitney Z = -1.52, p = 0.13), but the BPD and SoD groups differed significantly from the control and schizophrenia group (all Mann-Whitney *Z>* -1.98, all *p*<0.05). However data on educational level was only available for 6 patients in the SoD group and 51 patients in the schizophrenia group making this analysis on differences between groups difficult to interpret. See Table 1 for the distribution characteristics on age, sex, educational level, comorbidity and severity of depression as measured with the Beck’s Depression Inventory for each of the groups separately.

**Table 1:** Age, sex, and general face recognition performance for each of the four groups. P values are derived from overall group ANOVA highlighting significant differences across all groups.

	Schizophrenia	BPD	SoD	Healthy controls	*P*
	N = 57	N = 30	N = 25	N = 41	
**Age in years (SD)**	32.4 (9.2)	30.0 (8.9)	32.4 (9.2)	30.9 (8.5)	0.03
**Male:female ratio**	44:13	10:20	4:21	28:13	<0.001
Education (low; middle; high)*	15.7%; 51%; 33.3%	36.7%; 43.3%; 20%	66.7%; 33.3%; 0%	26.8%; 24.4%; 48.8%	0.01
PTSD comorbidity**	--	N = 6	N = 1	--	
BDI (SD)***	16.7 (12.5)	24.9 (10.9)	23.0 (9.9)	--	0.04
**#Correct Benton (SD)**	21.1 (2.3)	21.1 (1.7)	21.0 (1.9)	22.5 (1.9)	0.002

* Adjusted sample size due to missing data are N = 51 for schizophrenia; N = 30 for BPD; N = 6 for SoD; N = 41 for control groups.

** PTSD: Posttraumatic Stress Disorder.

*** BDI: Beck’s Depression Inventory, schizophrenia-sample from Delta Hospital only (N = 20); BPD and SoD groups did not differ on BDI, *F*(1,53) = 0.45, *p* = 0.51

### General Facial Recognition

Given the group differences on age, sex and educational level we considered adding these variables as covariates into the analyses of general face recognition. To examine whether these variables indeed influenced face recognition performance, we examined the relationship between Benton face recognition performance and both age and educational level using bivariate correlations as well as test for sex difference on Benton face recognition performance across the entire sample. There was no significant correlation between performance on Benton face recognition and age of participants, Pearson *r*(153) = 0.06, *p* = 0.43 or educational level, Spearman *rho*(128) = 0.05, *p* = 0.57 and male and female participants did not differ from each other on general face recognition, Mann-Whitney *Z* = -1.10, *p* = 0.27. We therefore did not add these variables as covariates to the subsequent analysis.

A one-way ANOVA on general face recognition revealed that the groups differed on the number of correct responses, *F*(3,149) = 5.06, *p* = 0.002. Bonferroni corrected post-hoc tests show that all patient groups; schizophrenia patients (*p* = 0.005), BPD patients (*p* = 0.02) and SoD patients (*p* = 0.02) performed worse than the control group. Patient groups did not differ from one another (all *p*>0.05). See Table 1 for the Benton facial recognition scores for each of the groups separately.

### Facial Affect Recognition

#### Errors

Four patients in the SoD group were excluded from the analysis as performance on the recognition of happy faces was considered an outlier (*Z*-score > 3). Similar as to the analysis on general face recognition, we considered adding the variables age, sex, educational level as well as general face recognition performance as covariates into the analyses of facial affect labeling. Correlational analyses between performance on facial affect labeling and age of participants, Pearson *r*(149) = -0.14, *p* = 0.09 or educational level, Spearman *rho*(128) = 0.16, *p* = 0.07 did not reach our α<0.05 threshold, and male and female participants did not differ from each other on facial affect labeling performance, Mann-Whitney *Z* = -1.42, *p* = 0.16. However, there was a significant correlation between performance on facial affect labeling and general face recognition, Pearson *r*(149) = 0.33, *p*<0.0001. We therefore included general face recognition performance as a covariate in subsequent analyses.

The 3x2x4 repeated measures within-subjects ANCOVA showed a significant interaction between Facial Affect*Group, *F*(5.74, 275,41) = 3.19, *p* = 0.005, suggesting that groups differ on labeling specific facial expressions. This interaction was also expressed in main effects of Facial Affect, *F*(1.91, 275,41) = 3.47, *p* = 0.04; Group, *F*(3,144) = 4.92, *p* = 0.03; and general face recognition, *F*(3,144) = 8.89, *p* = 0.003. The main effect of Emotional Intensity was not significant, *F*(1,144) = 0.55, *p* = 0.46, and no other (two or three-way) interactions were significant (all *p*>0.05).

Bonferroni adjusted post-hoc MANCOVA to delineate the Group*Facial Affect (combining 100% and 75% emotional intensity) interaction included all four face expressions, and thus also included neutral faces. This revealed no group differences on errors labeling happy facial expressions (all *p*>0.5). All following results are Bonferroni adjusted.

On labeling angry facial expressions, schizophrenia patients performed worse than non-psychiatric controls (*p* = 0.01), but similar to SoD patients (*p* = 0.16) and BPD patients (*p* = 0.99). However, BPD and SoD patients did not differ significantly from non-psychiatric controls (*p* = 0.18 and *p* = 0.99 respectively) or each other (*p* = 0.82). These data suggest that on labeling angry expressions schizophrenia patients performed worst and non-psychiatric controls performed best, with both the BPD and SoD groups performing in between schizophrenia patients and non-psychiatric controls.

On labeling fearful expressions, a close to similar pattern emerged. Schizophrenia patients performed worse than non-psychiatric controls (*p* = 0.005) and SoD patients (*p* = 0.04), but did not differ significantly from BPD patients (*p* = 0.99). BPD patients did not differ from non-psychiatric controls (*p* = 0.21) or SoD patients (p = 0.43), and SoD patients did not differ from non-psychiatric controls (p = 0.99). These data suggest that on labeling fearful expressions schizophrenia patients performed worse than non-psychiatric controls and SoD patients, with BPD patients performing in between schizophrenia patients and non-psychiatric controls/SoD patients.

**Fig 2:**
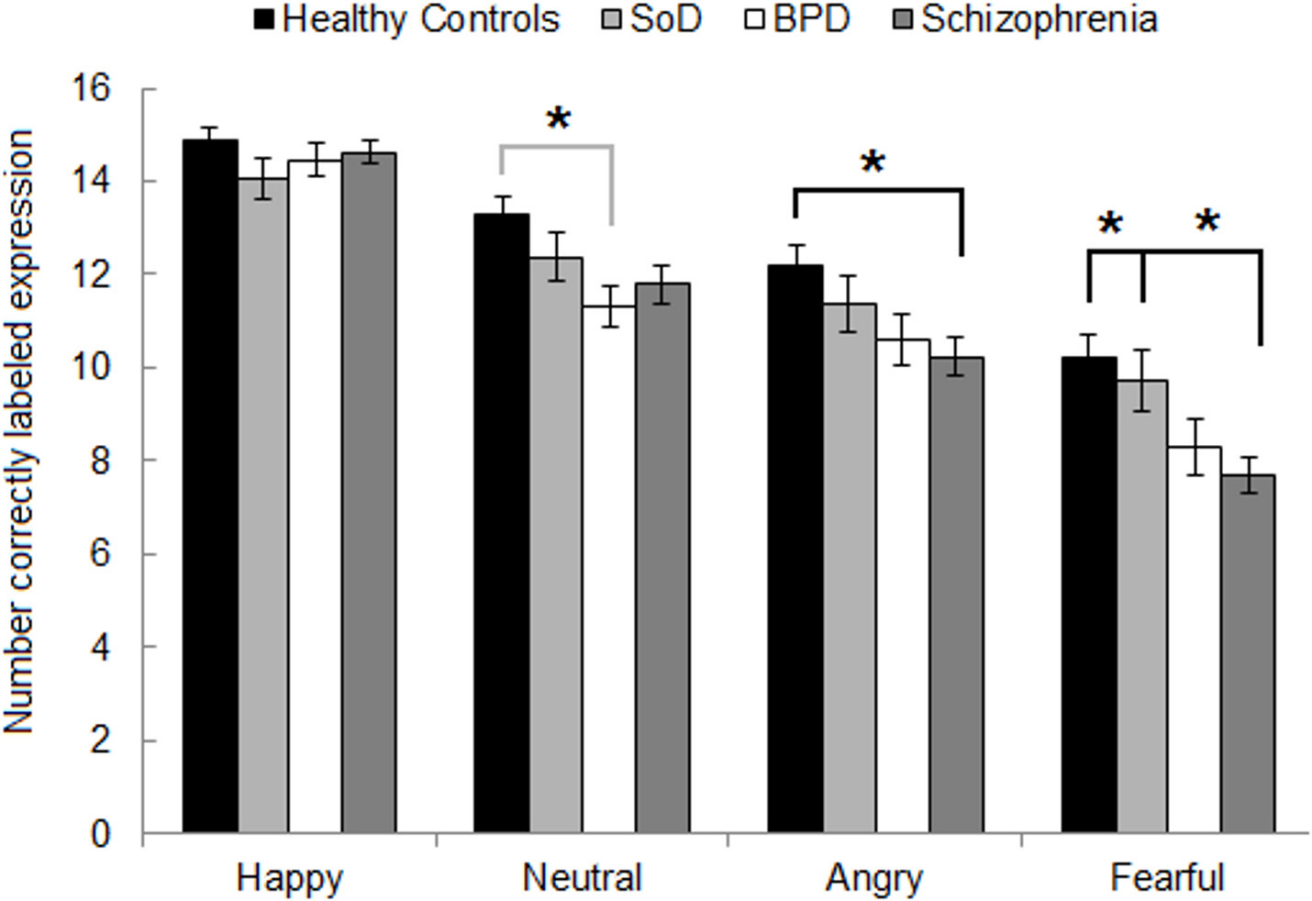
Number of correctly labeled faces (SEM) per emotion, per group. Asterisks reflect significant differences between groups: black for schizophrenia; grey for BPD.

On neutral expressions, BPD patients performed worse than non-psychiatric controls (p = 0.045). All other group comparisons were non-significant (all p>0.05). These data suggest that on labeling neutral expressions BPD patients performed worst and non-psychiatric controls performed best, with the schizophrenia and SoD groups performing in between. See Fig 2 for a graphic representation of the number of errors separated for each facial expression and each group.

#### Error pattern analyses

Given some group differences in labeling neutral, angry and fearful expressions we examined group differences in misattribution errors [66,67] for these expressions (previous analysis showed no group differences on emotional intensity, allowing combining 100% and 75% emotional intensity where applicable). Misattribution errors (%) are displayed in Table 2. Misattribution proportion scores (0–1) were calculated by dividing the number of misclassifications by the number of total errors made for that facial expression (e.g. neutral mistaken for fear/total mistakes labeling neutral). It should be noted that these analyses were on errors *only* and therefore group sizes differ depending on the group comparison [67]. Given the large amount of comparisons associated *p*-values were Bonferroni adjusted.

**Table 2:** Values represent percent misattribution errors for each of the four facial expressions in the degraded facial affect labeling task separated by group, e.g. 5.1% of happy misattributions were due to ‘Happy expressions being mistaken for Angry’ in schizophrenia patients. Bold values (along the diagonal) represent percent correct for each facial expression by group.

Actual emotion presented	Group	Answer given (%)			
		Happy	Angry	Fear	Neutral
**Happy**	SCZ	**93.05**	5.1	24.4	70.5
	BPD	**92.07**	4.4	32.6	63.0
	SoD	**87.65**	4.9	39.0	56.1
	HC	**90.04**	11.5	26.9	61.5
**Angry**	SCZ	10.2	**70.5**	41.1	48.7
	BPD	9.3	**72.07**	43.2	47.5
	SoD	9.2	**73.79**	51.7	39.1
	HC	5.0	**74.17**	40.3	54.7
**Fear**	SCZ	10.4	13.1	**57.84**	76.5
	BPD	8.2	16.9	**60.17**	74.9
	SoD	12.5	11.7	**61.45**	75.8
	HC	7.9	5.9	**56.09**	86.1
**Neutral**	SCZ	66.7	22.1	11.3	**78.61**
	BPD	33.3	34.8	31.9	**75.69**
	SoD	52.6	26.1	21.1	**77.11**
	HC	60.9	25.5	13.6	**79.7**

SCZ: schizophrenia; BPD: borderline personality disorder; SoD: somatoform disorder; HC: healthy controls.

Kruskal Wallis test on all possible misattributions (9 total: each of 3 facial expressions could be mistaken for three expressions) showed overall group differences in mistaking neutral expressions for happy (*Chi-Square* = 17.97, *df* = 3, *p*<0.0001) and fear (*Chi-Square* = 16.14, *df* = 3, *p* = 0.001). All other misattributions were non-significant based on Bonferroni adjusted α = 0.006. Further non-parametric Whitney-Mann U independent sample tests (Bonferroni adjusted α = 0.004 based on 12 comparisons: 4 groups; 2 errors (happy instead of neutral; fear instead of neutral)) demonstrated that BPD patients mistook neutral expressions more often for fearful compared to schizophrenia patients, *Z* = -3.89, *p* = 0.0001, and non-psychiatric controls, *Z* = -2.86, *p* = 0.004. As a consequence, BPD patients mistook neutral expression less often for happy as compared to schizophrenia patients, *Z* = -4.16, *p* = 0.0003, who mistook neutral expressions most often for happy. All other comparisons *p*>0.004.

## Discussion

The aim of the present study was to investigate facial affect labeling in patients with BPD and schizophrenia, and to contrast their performance with that of patients with SoD and healthy controls. The question of differential impairment in facial affect recognition in BPD compared to that in psychotic and other psychiatric disorders may point towards a clearer delineation of the similarities and differences in these disorders with regard to a key factor in psychopathology, emotion processing.

Given that the recognition of faces is a complex cognitive process, we first evaluated general face recognition ability in all patients groups. Results show that all patient groups, including SoD patients, made more errors in general face recognition compared to the non-psychiatric control group. In fact, there were no differences between the three patient groups on general face recognition accuracy. The presence of general face processing difficulties in all three patient groups suggests that having a psychiatric disorder may negatively influence the ability to recognize faces. Although face processing deficits have been observed in schizophrenia [68, 69], this is novel for BPD and SoD. It remains unclear whether the presence of general face processing deficits is due to deficits in the processing of faces per se or is due to generally impaired perceptual [70] or cognitive functioning, such as attention deficits or error monitoring in psychiatric patients [71, 72]. We further observed that general face recognition significantly predicted facial affect labeling performance, i.e., individuals who made more errors in general face recognition also made more errors in facial affect labeling. Therefore, general face recognition performance was included as a covariate in our analyses of facial affect labeling.

Our results on facial affect labeling show that patients with schizophrenia made more errors in the labeling of fearful and angry facial expressions than non-psychiatric control participants, and more errors in labeling fearful expressions compared to psychiatric controls (SoD). These findings are consistent with our first hypothesis, that schizophrenia would be associated with deficits in identification of negative affect in facial expressions. Error pattern analyses however demonstrate that schizophrenia patients made similar misattribution errors as non-psychiatric and psychiatric controls (SoD patients). However, compared to BPD patients, schizophrenia patients were more likely to mistake neutral faces for happy even though they performed comparably to all other groups on labeling neutral faces overall. This suggests that schizophrenia can be associated with the more typical positively valenced distortions in ambiguous (e.g. neutral) facial affect processing compared to BPD, despite an inaccuracy in identifying negative affect in facial expressions compared to controls. This is consistent with the view that in schizophrenia emotion deficits are not limited to distortions related to a generally dysphoric outlook, but to broader impairments in social-emotional processing including affective mentalizing [73].

BPD was associated with more errors in the labeling of neutral expressions, but not fearful or angry expressions as compared to non-psychiatric controls. We also did not observe significant differences between BPD patients and psychiatric controls on labeling angry, fearful or neutral expressions. Although there were similarities in facial affect labeling impairments for patients with schizophrenia and BPD (as evident in the absence of significant differences in direct comparisons between BPD and schizophrenia patients), the misattribution errors made by BPD patients primarily involved a dysphoric distortion of affectively neutral facial displays. BPD patients mistook neutral expressions more often for fearful compared schizophrenia patients as well as healthy controls. The finding in BPD is in line with previous studies reporting a bias to interpret ambiguous facial expressions in a negative way or the magnification of subtle expression of (negative) affect [17–20]. This projection of negative affect corroborates findings from prior studies that show that BPD is associated with enhanced sensitivity to fear-related interpersonal stimuli, which could be related to the observed emotion dysregulation in BPD [8, 74–76].

Our finding that patients with BPD mislabeled neutral expressions for fearful is worth noting as it extends a prior report demonstrating a bias perceiving anger [e.g. 18]. That said, results of an fMRI study reports neural deactivations in response to fearful, but not anger faces [77]. Specifically, they observed a significantly larger magnitude of deactivation in the bilateral rostral/subgenual anterior cingulate cortex (ACC) to fear in people diagnosed with BPD relative to healthy controls. Moreover when fearful faces were compared to neutral faces they found deactivation in left ACC and amygdala in BPD.

As expected, SoD patients did not differ in facial affect labeling performance or misattribution errors from non-psychiatric control participants, but unexpectedly also did not differ significantly from BPD and schizophrenia patients (except for labeling fearful faces). This suggests that SoD patients performed in between controls and schizophrenia/BPD patients and supports the idea of (relatively) minor facial affect processing deficits in SoD [37]. This is noteworthy as SoD patients did perform worse on general face recognition than non-psychiatric control participants. Taken together, these data are consistent with the view that, although deficits in facial recognition per se may be generally associated with psychiatric disorders, visual social-emotional deficits in facial affect labeling appear to be more strongly related to BPD and schizophrenia as tested here.

Our observation of facial affect labeling impairments in BPD and schizophrenia patients could suggest that the two disorders share some common neuroanatomical basis. A meta-analysis of functional neuroimaging studies on emotion perception in schizophrenia summarized reduced activation in brain regions associated with emotion processing such as the amygdala, anterior cingulate cortex, and regions in prefrontal cortex [78]. It has been suggested that the amygdala might by chronically hyperactive in schizophrenia, leading to the impression of reduced activation in fMRI designs in which activation for emotional faces is compared to activation for neutral faces [79, 80]. That is, when baseline amygdala activation is higher in schizophrenia patients, the contrast between emotional and neutral stimuli will not yield additional activation. With respect to BPD, prior research has reported increased activation in the amygdala in response to viewing negatively valenced pictures [81] and facial affect [61,77] as compared to control participants. Cumulative evidence for a dysfunction in a frontolimbic network to underlie borderline symptoms has further been recognized [82]. Although our study cannot provide conclusive evidence, amygdala dysregulation might contribute to facial affect processing deficits in schizophrenia and BPD. Further studies should directly compare patients with schizophrenia and BPD using functional neuroimaging simultaneously with facial affect recognition.

As expected, none of the groups differed on the labeling of happy faces. Typically the detection of happy facial expressions is the easiest (possibly due to a pop-out effect), and emotions such as anger and fear are much harder to recognize [24]. We further observed no significant effect of increased emotional intensity on facial affect labeling performance, which was surprising but likely due to inclusion of general face recognition performance as a covariate in our analyses.

Overall, these findings extend those of previous studies that studied facial affect processing in other psychiatric samples, by showing evidence of deficits in facial affect labeling in schizophrenia and BPD and potentially SoD, which requires future research. Treatment for BPD thus may benefit from including affect recognition training similar to that which has proven successful for patients with schizophrenia [83]. On the other hand, the more specifically negative-valenced impairments in facial affect labeling observed in schizophrenia patients may indicate schizophrenia treatment to be enhanced by including skills-training that has shown to improve affective and interpersonal regulation in patients with chronic affect regulation problems (e.g. [84]) as well as those specifically diagnosed with BPD [6]. Recent findings that emotion-based training with the addition of oxytocin proved to improve facial affect labeling suggest that targeted biological interventions may have utility as well [85], although this might be limited to more difficult, higher-order social cognitive tasks [86].

Limitations of the study include that the groups were not fully matched on demographic variables (age, sex and educational level based on available data) and no data was available on intellectual functioning, illness duration or a measure of symptom severity applicable to all three clinical groups. Although statistical analyses on associations between various demographic variables and general face/facial affect performance were non-significant, it is possible that other clinical differences between the groups may account for differences in task performance on both experimental tasks. Similarly, it is possible that the various types of medication used by the three clinical groups influenced task performances. A second limitation is that the number of errors made in the facial affect labeling task was relatively small for happy facial expressions as well as certain misattribution categories (e.g. mislabeling a happy face for angry or vice versa). Hence, our analyses on misattribution errors between groups should be interpreted with some caution and requires further scientific exploration and repetition. Thirdly, our task design incorporated unrestricted response times and facial stimuli were shown indefinitely until a response was made. Although this was purposefully done in order to capture facial affect labeling differences without effects attributable to response speed typically lower in those with schizophrenia, this reduces the external validity of the task as expressions encountered in real life are revealed more rapidly. Moreover, we cannot rule out that the degrading of facial affect stimuli in this task has had differential effects on labeling any of the emotional expressions presented.

Despite these considerations, our study represents one of the few attempts to disentangle possible similarities and differences in facial affect processing in various psychiatric patients. Our findings suggest that schizophrenia and BPD patients demonstrate partly unique facial affect labeling errors and a specific negative misattribution of ambiguous facial expressions in BPD. This could have implications for the psychobiological study and treatment of these disorders.

## Supporting Information

S1 File SI_raw data face processing.sav (SAV)
